# 6-Allyl-3-(6-chloro-3-pyridylmeth­yl)-6,7-dihydro-3*H*-1,2,3-triazolo[4,5-*d*]pyrimidin-7-imine

**DOI:** 10.1107/S1600536809048168

**Published:** 2009-11-25

**Authors:** Dong-Feng Pan, Jing Xu, Jun-Kai Ma, Hong Luo, Zuan Ma

**Affiliations:** a Department of Oncology, Renmin Hospital, Yunyang Medical College, Shiyan, 442000, People’s Republic of China; b Institute of Medicinal Chemistry, Yunyang Medical College, Shiyan, 442000, People’s Republic of China

## Abstract

The title compound, C_13_H_12_ClN_7_, crystallizes with two independent mol­ecules in the asymmetric unit, each with similar geometries. The dihedral angles between the triazole and pyrimidine rings are 0.45 (9) and 1.00 (10)° in the two mol­ecules. A number of N—H⋯N hydrogen bonds co-operate with C–H⋯N contacts, forming a supra­molecular array in the *ab* plane. C—H⋯π inter­actions are also present. One of the vinyl groups was found to be disordered so that the C(H)=CH_2_ atoms were resolved over two positions with the major component having a site occupancy factor of 0.539 (4).

## Related literature

For general background to 8-aza­purine derivatives, see: Albert (1986 ▶). For the biological activity of 8-aza­purines, see: Shiokawa *et al.* (1986 ▶); Slusarkchyk & Zahler (1989 ▶); Subramanian & Gerwick (1989 ▶); Vince & Hua (1990 ▶); Yamamoto *et al.* (1994 ▶).
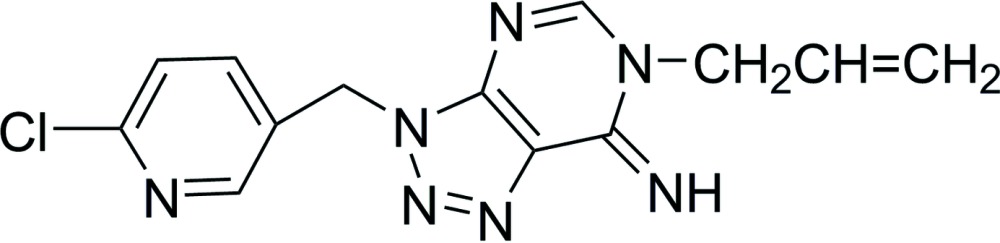


## Experimental

### 

#### Crystal data

C_13_H_12_ClN_7_
*M* *_r_* = 301.75
Triclinic, 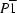

*a* = 7.2845 (7) Å
*b* = 13.2684 (12) Å
*c* = 14.7069 (14) Å
α = 87.351 (1)°
β = 81.752 (1)°
γ = 82.917 (1)°
*V* = 1395.4 (2) Å^3^
*Z* = 4
Mo *K*α radiation
μ = 0.28 mm^−1^
*T* = 296 K
0.48 × 0.46 × 0.43 mm

#### Data collection

Bruker SMART APEX CCD area-detector diffractometer
Absorption correction: none
10116 measured reflections
5052 independent reflections
4277 reflections with *I* > 2σ(*I*)
*R*_int_ = 0.015

#### Refinement

*R*[*F* ^2^ > 2σ(*F* ^2^)] = 0.040
*wR*(*F* ^2^) = 0.109
*S* = 1.03
5052 reflections
384 parameters
22 restraints
H-atom parameters constrained
Δρ_max_ = 0.35 e Å^−3^
Δρ_min_ = −0.36 e Å^−3^

### 

Data collection: *SMART* (Bruker, 2000 ▶); cell refinement: *SAINT* (Bruker, 2000 ▶); data reduction: *SAINT*; program(s) used to solve structure: *SHELXS97* (Sheldrick, 2008 ▶); program(s) used to refine structure: *SHELXL97* (Sheldrick, 2008 ▶); molecular graphics: *SHELXTL* (Sheldrick, 2008 ▶); software used to prepare material for publication: *SHELXTL*.

## Supplementary Material

Crystal structure: contains datablocks global, I. DOI: 10.1107/S1600536809048168/tk2573sup1.cif

Structure factors: contains datablocks I. DOI: 10.1107/S1600536809048168/tk2573Isup2.hkl

Additional supplementary materials:  crystallographic information; 3D view; checkCIF report

## Figures and Tables

**Table 1:** Hydrogen-bond geometry (Å, °)

*D*—H⋯*A*	*D*—H	H⋯*A*	*D*⋯*A*	*D*—H⋯*A*
N10—H10*A*⋯N1	0.86	2.44	3.292 (3)	173
N3—H3*A*⋯N8^i^	0.86	2.45	3.299 (2)	169
C11—H11*A*⋯N3	0.97	2.41	2.749 (2)	100
C19—H19⋯N11	0.93	2.59	2.909 (3)	101
C3—H3⋯N13	0.93	2.46	3.309 (2)	151
C11—H11*A*⋯*Cg*4^ii^	0.97	2.87	3.446 (2)	119
C24—H24*A*⋯*Cg*1^iii^	0.97	2.99	3.851 (3)	149

Symmetry codes: (i) 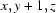; (ii) 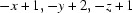; (iii) 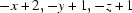. *Cg*4 and *Cg*1 are the centroids of the N4–N6/C1/C4 and N11–N13/C14/C17C4 rings, respectively.

**Fig. 1.**
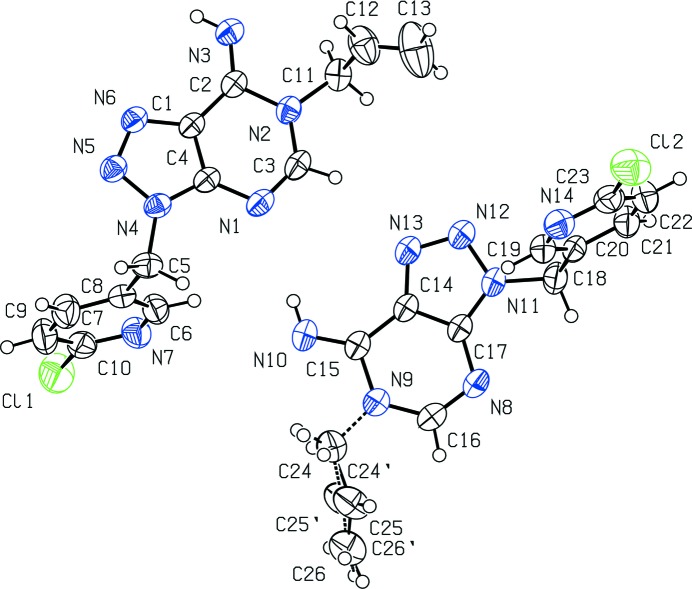
The molecular structures of the two independent molecules in (I), showing 50% probability displacement ellipsoids and the atom-numbering scheme.

**Fig. 2.**
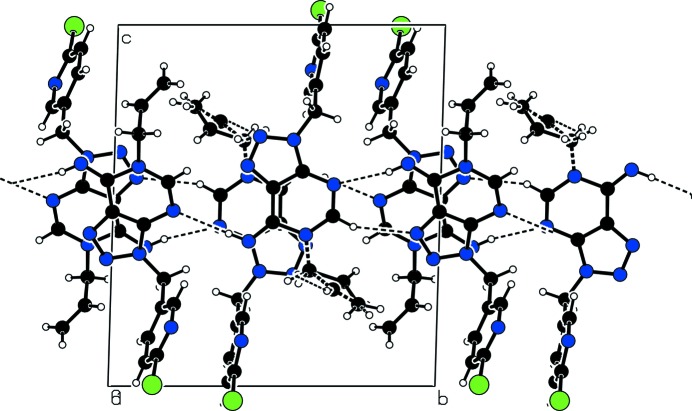
A view of a supramolecular layer in (I). Hydrogen bonds are shown as dashed lines.

## supplementary crystallographic information

### Comment

1,2,3-Triazolo[4,5-*d*]pyrimidines, *i.e.* 8-azapurines (Albert, 1986), have attracted attention because some of these derivatives exhibit anti-viral (Slusarkchyk & Zahler, 1989), anti-tumour (Slusarkchyk & Zahler, 1989; Vince & Hua, 1990), and herbicidal activities (Subramanian & Gerwick, 1989). Neonicotinoid insecticides, as nicotinic acetylcholine receptor inhibitors, have also attracted increasing attention because of their low toxicity, wide range of activities, and high potency (Shiokawa *et al.*, 1986). It has been found that most biologically active nicotinic compounds contain the 3-aminomethylpyridine group (Yamamoto *et al.*, 1994). Herein, we report the crystal structure of (I), Fig. 1, which was synthesized by introducing pyridine rings into a 1,2,3-triazolo[4,5-*d*]pyrimidine framework.

Several N—H···N hydrogen bonding contacts, together with C—H···N and C—H···π interactions, lead to the formation of supramolecular arrays in the *ab* plane, Table 1 and Fig. 2. In addition π—π stacking interactions stabilize the crystal structure, with the shortest centroid-centroid distance of 3.412 (1) Å occurring between centrosymmetrically related planes through the (N4–N6, C1, C4) rings, symmetry operation: 2-x, 1-y, 1-z.

### Experimental

Allylamine (1 mmol) in anhydrous acetonitrile (4 ml) was added dropwise to a solution of ethyl-*N*-3-((6-chloropyridin-3-yl)methyl)-5-cyano-3*H*-1,2,3- triazol-4-yl-formimidate (1 mmol) in anhydrous acetonitrile (8 ml) at room temperature. The mixture was stirred until the reaction was complete (by thin layer chromatography) and the solution concentrated under vacuum. The residue was recrystallized from anhydrous ethanol to give (I) (yield 87%). Colourless crystals were grown from a dichloromethane and petroleum ether (1:3 *v*/*v*) solution of (I).

### Refinement

H atoms were placed in calculated positions, with C—H distances in the range 0.93–0.97 Å and N—H distances of 0.86 Å, and included in the final cycles of refinement using a riding-model approximation, with *U*_iso_(H) = 1.2–1.5*U*_eq_(carrier atom). A rotating group model was used for the methyl groups. Disorder was noted in the C24-C26 vinyl substituent in that two positions were resolved for the C25 atom. From refinement, the major component had a site occupancy factor = 0.539 (4).

### Crystal data

**Table:**

C_13_H_12_ClN_7_	*Z* = 4
*M**_r_* = 301.75	*F*(000) = 624
Triclinic, *P*1	*D*_x_ = 1.436 Mg m^−^^3^
Hall symbol: -P 1	Mo *K*α radiation, λ = 0.71073 Å
*a* = 7.2845 (7) Å	Cell parameters from 5848 reflections
*b* = 13.2684 (12) Å	θ = 2.8–28.2°
*c* = 14.7069 (14) Å	µ = 0.28 mm^−^^1^
α = 87.351 (1)°	*T* = 296 K
β = 81.752 (1)°	Block, colorless
γ = 82.917 (1)°	0.48 × 0.46 × 0.43 mm
*V* = 1395.4 (2) Å^3^	

### Data collection

**Table:**

Bruker SMART APEX CCD area-detector diffractometer	4277 reflections with *I* > 2σ(*I*)
Radiation source: fine-focus sealed tube	*R*_int_ = 0.015
graphite	θ_max_ = 25.5°, θ_min_ = 2.8°
φ and ω scans	*h* = −8→8
10116 measured reflections	*k* = −15→16
5052 independent reflections	*l* = −17→17

### Refinement

**Table:**

Refinement on *F*^2^	Secondary atom site location: difference Fourier map
Least-squares matrix: full	Hydrogen site location: inferred from neighbouring sites
*R*[*F*^2^ > 2σ(*F*^2^)] = 0.040	H-atom parameters constrained
*wR*(*F*^2^) = 0.109	*w* = 1/[σ^2^(*F*_o_^2^) + (0.0474*P*)^2^ + 0.5781*P*] where *P* = (*F*_o_^2^ + 2*F*_c_^2^)/3
*S* = 1.03	(Δ/σ)_max_ = 0.001
5052 reflections	Δρ_max_ = 0.35 e Å^−^^3^
384 parameters	Δρ_min_ = −0.36 e Å^−^^3^
22 restraints	Extinction correction: *SHELXL*, Fc^*^=kFc[1+0.001xFc^2^λ^3^/sin(2θ)]^-1/4^
Primary atom site location: structure-invariant direct methods	Extinction coefficient: 0.050 (2)

### Special details

**Table:**

Geometry. All e.s.d.'s (except the e.s.d. in the dihedral angle between two l.s. planes)are estimated using the full covariance matrix. The cell e.s.d.'s are takeninto account individually in the estimation of e.s.d.'s in distances, anglesand torsion angles; correlations between e.s.d.'s in cell parameters are onlyused when they are defined by crystal symmetry. An approximate (isotropic)treatment of cell e.s.d.'s is used for estimating e.s.d.'s involving l.s. planes.
Refinement. Refinement of *F*^2^ against ALL reflections. The weighted *R*-factor *wR* andgoodness of fit *S* are based on *F*^2^, conventional *R*-factors *R* are basedon *F*, with *F* set to zero for negative *F*^2^. The threshold expression of*F*^2^ > σ(*F*^2^) is used only for calculating *R*-factors(gt) *etc*. and isnot relevant to the choice of reflections for refinement. *R*-factors basedon *F*^2^ are statistically about twice as large as those based on *F*, and *R*-factors based on ALL data will be even larger.

### Fractional atomic coordinates and isotropic or equivalent isotropic displacement parameters (Å^2^)

**Table:**

	*x*	*y*	*z*	*U*_iso_*/*U*_eq_	Occ. (<1)
C24	0.7964 (4)	0.39425 (19)	0.67973 (16)	0.0710 (7)	0.539 (4)
H24A	0.9002	0.4288	0.6913	0.085*	0.539 (4)
H24B	0.6844	0.4265	0.7159	0.085*	0.539 (4)
C25	0.8281 (6)	0.2896 (3)	0.7111 (3)	0.0666 (9)	0.539 (4)
H25	0.9341	0.2520	0.6814	0.080*	0.539 (4)
C26	0.7319 (5)	0.2418 (3)	0.7732 (2)	0.1002 (10)	0.539 (4)
H26A	0.6239	0.2747	0.8058	0.120*	0.539 (4)
H26B	0.7692	0.1738	0.7864	0.120*	0.539 (4)
C24'	0.7964 (4)	0.39425 (19)	0.67973 (16)	0.0710 (7)	0.461 (4)
H24C	0.9279	0.3756	0.6840	0.085*	0.461 (4)
H24D	0.7600	0.4593	0.7086	0.085*	0.461 (4)
C25'	0.6943 (8)	0.3207 (4)	0.7325 (3)	0.0666 (9)	0.461 (4)
H25'	0.5659	0.3385	0.7363	0.080*	0.461 (4)
C26'	0.7319 (5)	0.2418 (3)	0.7732 (2)	0.1002 (10)	0.461 (4)
H26C	0.8561	0.2159	0.7746	0.120*	0.461 (4)
H26D	0.6368	0.2068	0.8034	0.120*	0.461 (4)
C1	0.7678 (2)	1.00162 (12)	0.51743 (12)	0.0346 (4)	
C2	0.7015 (2)	1.02006 (12)	0.42960 (12)	0.0360 (4)	
C3	0.7187 (3)	0.83395 (13)	0.43489 (14)	0.0448 (4)	
H3	0.6996	0.7774	0.4040	0.054*	
C4	0.8029 (2)	0.90450 (12)	0.55236 (12)	0.0362 (4)	
C5	0.9108 (3)	0.83866 (14)	0.70333 (13)	0.0457 (4)	
H5A	1.0253	0.8510	0.7252	0.055*	
H5B	0.9309	0.7719	0.6768	0.055*	
C6	0.5880 (3)	0.80588 (16)	0.77284 (13)	0.0493 (5)	
H6	0.5802	0.7758	0.7179	0.059*	
C7	0.7544 (3)	0.84151 (13)	0.78275 (12)	0.0406 (4)	
C8	0.7665 (3)	0.88164 (17)	0.86595 (15)	0.0588 (5)	
H8	0.8770	0.9047	0.8763	0.071*	
C9	0.6143 (3)	0.88762 (18)	0.93401 (15)	0.0639 (6)	
H9	0.6193	0.9140	0.9910	0.077*	
C10	0.4556 (3)	0.85311 (15)	0.91408 (13)	0.0511 (5)	
C11	0.6112 (3)	0.92790 (16)	0.30226 (13)	0.0484 (5)	
H11A	0.5276	0.9899	0.2960	0.058*	
H11B	0.5401	0.8711	0.3001	0.058*	
C12	0.7634 (3)	0.9228 (2)	0.22394 (16)	0.0681 (6)	
H12	0.8453	0.9719	0.2193	0.082*	
C13	0.7911 (4)	0.8556 (3)	0.1618 (2)	0.1080 (12)	
H13A	0.7121	0.8053	0.1641	0.130*	
H13B	0.8902	0.8573	0.1144	0.130*	
C14	0.7367 (2)	0.51225 (13)	0.45004 (13)	0.0407 (4)	
C15	0.7607 (3)	0.51058 (14)	0.54603 (13)	0.0434 (4)	
C16	0.7703 (3)	0.32703 (15)	0.52893 (15)	0.0523 (5)	
H16	0.7816	0.2638	0.5589	0.063*	
C17	0.7361 (2)	0.42382 (13)	0.40561 (13)	0.0398 (4)	
C18	0.7094 (3)	0.39121 (15)	0.23947 (14)	0.0514 (5)	
H18A	0.5996	0.4168	0.2114	0.062*	
H18B	0.6960	0.3220	0.2611	0.062*	
C19	1.0541 (3)	0.40537 (14)	0.18782 (14)	0.0499 (5)	
H19	1.0650	0.4172	0.2486	0.060*	
C20	0.8801 (3)	0.39059 (13)	0.16747 (13)	0.0440 (4)	
C21	0.8673 (3)	0.37205 (16)	0.07674 (14)	0.0549 (5)	
H21	0.7530	0.3618	0.0598	0.066*	
C22	1.0239 (3)	0.36883 (17)	0.01182 (15)	0.0608 (6)	
H22	1.0184	0.3558	−0.0493	0.073*	
C23	1.1885 (3)	0.38548 (15)	0.04051 (15)	0.0546 (5)	
Cl1	0.25485 (10)	0.86518 (6)	0.99613 (4)	0.0824 (2)	
Cl2	1.39020 (10)	0.38267 (5)	−0.04027 (5)	0.0812 (2)	
N1	0.7805 (2)	0.81572 (11)	0.51360 (11)	0.0446 (4)	
N2	0.6786 (2)	0.92572 (11)	0.39244 (10)	0.0399 (3)	
N3	0.6629 (2)	1.10214 (11)	0.38456 (11)	0.0450 (4)	
H3A	0.6766	1.1591	0.4069	0.054*	
N4	0.86503 (19)	0.91590 (10)	0.63304 (10)	0.0384 (3)	
N5	0.8663 (2)	1.01637 (11)	0.64796 (10)	0.0412 (4)	
N6	0.8078 (2)	1.06876 (11)	0.57735 (10)	0.0386 (3)	
N7	0.4377 (2)	0.81185 (14)	0.83706 (11)	0.0541 (4)	
N8	0.7516 (2)	0.32692 (11)	0.44197 (12)	0.0503 (4)	
N9	0.7752 (2)	0.40853 (12)	0.58091 (11)	0.0487 (4)	
N10	0.7707 (3)	0.58234 (13)	0.59890 (12)	0.0575 (5)	
H10A	0.7618	0.6440	0.5777	0.069*	
N11	0.7175 (2)	0.45313 (11)	0.31812 (11)	0.0451 (4)	
N12	0.7058 (3)	0.55636 (12)	0.30882 (12)	0.0545 (4)	
N13	0.7175 (3)	0.59204 (12)	0.38966 (12)	0.0515 (4)	
N14	1.2090 (2)	0.40379 (13)	0.12549 (12)	0.0551 (4)	

### Atomic displacement parameters (Å^2^)

**Table:**

	*U*^11^	*U*^22^	*U*^33^	*U*^12^	*U*^13^	*U*^23^
C24	0.0985 (19)	0.0654 (15)	0.0573 (14)	−0.0258 (13)	−0.0265 (13)	0.0029 (11)
C25	0.065 (2)	0.079 (2)	0.0500 (19)	0.0091 (18)	−0.0045 (17)	0.0038 (16)
C26	0.150 (3)	0.093 (2)	0.0610 (17)	−0.037 (2)	−0.0126 (18)	0.0071 (16)
C24'	0.0985 (19)	0.0654 (15)	0.0573 (14)	−0.0258 (13)	−0.0265 (13)	0.0029 (11)
C25'	0.065 (2)	0.079 (2)	0.0500 (19)	0.0091 (18)	−0.0045 (17)	0.0038 (16)
C26'	0.150 (3)	0.093 (2)	0.0610 (17)	−0.037 (2)	−0.0126 (18)	0.0071 (16)
C1	0.0295 (8)	0.0307 (8)	0.0413 (9)	−0.0047 (6)	0.0045 (7)	−0.0034 (7)
C2	0.0276 (8)	0.0352 (9)	0.0432 (10)	−0.0045 (6)	0.0037 (7)	−0.0052 (7)
C3	0.0447 (10)	0.0334 (9)	0.0554 (12)	−0.0065 (7)	0.0007 (8)	−0.0110 (8)
C4	0.0312 (8)	0.0321 (8)	0.0428 (10)	−0.0045 (6)	0.0042 (7)	−0.0023 (7)
C5	0.0446 (10)	0.0416 (10)	0.0485 (11)	−0.0001 (8)	−0.0046 (8)	0.0060 (8)
C6	0.0538 (11)	0.0573 (12)	0.0388 (10)	−0.0159 (9)	−0.0041 (8)	−0.0049 (9)
C7	0.0466 (10)	0.0334 (9)	0.0414 (10)	−0.0049 (7)	−0.0070 (8)	0.0050 (7)
C8	0.0591 (13)	0.0651 (14)	0.0568 (13)	−0.0206 (10)	−0.0102 (10)	−0.0094 (10)
C9	0.0783 (15)	0.0719 (15)	0.0449 (12)	−0.0233 (12)	−0.0028 (11)	−0.0168 (10)
C10	0.0619 (12)	0.0475 (11)	0.0421 (11)	−0.0126 (9)	0.0040 (9)	0.0009 (9)
C11	0.0442 (10)	0.0509 (11)	0.0512 (11)	−0.0049 (8)	−0.0076 (8)	−0.0114 (9)
C12	0.0555 (13)	0.1005 (19)	0.0509 (13)	−0.0218 (12)	−0.0025 (10)	−0.0115 (12)
C13	0.0756 (18)	0.169 (3)	0.0780 (19)	−0.0095 (19)	0.0080 (15)	−0.055 (2)
C14	0.0416 (9)	0.0328 (9)	0.0478 (10)	−0.0046 (7)	−0.0048 (8)	−0.0076 (8)
C15	0.0442 (10)	0.0377 (9)	0.0494 (11)	−0.0077 (8)	−0.0063 (8)	−0.0065 (8)
C16	0.0639 (13)	0.0353 (10)	0.0589 (13)	−0.0094 (9)	−0.0093 (10)	−0.0001 (9)
C17	0.0397 (9)	0.0332 (9)	0.0464 (11)	−0.0049 (7)	−0.0040 (8)	−0.0070 (7)
C18	0.0596 (12)	0.0466 (11)	0.0516 (12)	−0.0104 (9)	−0.0122 (9)	−0.0133 (9)
C19	0.0639 (13)	0.0438 (11)	0.0448 (11)	−0.0080 (9)	−0.0150 (9)	−0.0042 (8)
C20	0.0581 (11)	0.0306 (9)	0.0455 (11)	−0.0040 (8)	−0.0139 (9)	−0.0054 (7)
C21	0.0660 (13)	0.0504 (12)	0.0518 (12)	−0.0074 (10)	−0.0178 (10)	−0.0096 (9)
C22	0.0794 (16)	0.0599 (13)	0.0441 (12)	−0.0061 (11)	−0.0110 (11)	−0.0102 (10)
C23	0.0660 (13)	0.0419 (11)	0.0532 (13)	−0.0013 (9)	−0.0040 (10)	0.0004 (9)
Cl1	0.0861 (5)	0.0912 (5)	0.0643 (4)	−0.0290 (4)	0.0286 (3)	−0.0165 (3)
Cl2	0.0782 (4)	0.0853 (5)	0.0717 (4)	−0.0018 (3)	0.0105 (3)	0.0013 (3)
N1	0.0481 (9)	0.0305 (8)	0.0540 (10)	−0.0051 (6)	−0.0016 (7)	−0.0038 (7)
N2	0.0381 (8)	0.0369 (8)	0.0441 (8)	−0.0049 (6)	−0.0014 (6)	−0.0074 (6)
N3	0.0471 (9)	0.0364 (8)	0.0506 (9)	−0.0031 (7)	−0.0054 (7)	−0.0007 (7)
N4	0.0371 (8)	0.0338 (7)	0.0424 (8)	−0.0049 (6)	0.0010 (6)	0.0006 (6)
N5	0.0408 (8)	0.0376 (8)	0.0442 (9)	−0.0071 (6)	0.0002 (6)	−0.0029 (7)
N6	0.0379 (8)	0.0336 (7)	0.0431 (8)	−0.0062 (6)	0.0008 (6)	−0.0028 (6)
N7	0.0555 (10)	0.0642 (11)	0.0438 (9)	−0.0203 (8)	0.0004 (8)	−0.0022 (8)
N8	0.0652 (11)	0.0330 (8)	0.0537 (10)	−0.0085 (7)	−0.0071 (8)	−0.0074 (7)
N9	0.0599 (10)	0.0410 (9)	0.0482 (9)	−0.0128 (7)	−0.0117 (8)	−0.0012 (7)
N10	0.0796 (12)	0.0424 (9)	0.0541 (10)	−0.0111 (8)	−0.0146 (9)	−0.0116 (8)
N11	0.0544 (9)	0.0351 (8)	0.0473 (9)	−0.0064 (7)	−0.0082 (7)	−0.0094 (7)
N12	0.0752 (12)	0.0367 (9)	0.0527 (10)	−0.0044 (8)	−0.0139 (9)	−0.0055 (7)
N13	0.0693 (11)	0.0337 (8)	0.0525 (10)	−0.0035 (7)	−0.0124 (8)	−0.0076 (7)
N14	0.0595 (11)	0.0495 (10)	0.0570 (11)	−0.0068 (8)	−0.0114 (9)	0.0004 (8)

### Geometric parameters (Å, °)

**Table:**

C24—C25	1.445 (5)	C11—H11B	0.9700
C24—N9	1.485 (3)	C12—C13	1.283 (4)
C24—H24A	0.9700	C12—H12	0.9300
C24—H24B	0.9700	C13—H13A	0.9300
C25—C26	1.265 (5)	C13—H13B	0.9300
C25—H25	0.9300	C14—N13	1.357 (2)
C26—H26A	0.9300	C14—C17	1.370 (2)
C26—H26B	0.9300	C14—C15	1.446 (3)
C25'—H25'	0.9300	C15—N10	1.272 (2)
C1—N6	1.366 (2)	C15—N9	1.423 (2)
C1—C4	1.373 (2)	C16—N8	1.305 (3)
C1—C2	1.443 (2)	C16—N9	1.360 (2)
C2—N3	1.271 (2)	C16—H16	0.9300
C2—N2	1.426 (2)	C17—N11	1.348 (2)
C3—N1	1.303 (3)	C17—N8	1.367 (2)
C3—N2	1.361 (2)	C18—N11	1.461 (2)
C3—H3	0.9300	C18—C20	1.512 (3)
C4—N4	1.350 (2)	C18—H18A	0.9700
C4—N1	1.369 (2)	C18—H18B	0.9700
C5—N4	1.465 (2)	C19—N14	1.346 (3)
C5—C7	1.509 (3)	C19—C20	1.383 (3)
C5—H5A	0.9700	C19—H19	0.9300
C5—H5B	0.9700	C20—C21	1.386 (3)
C6—N7	1.337 (3)	C21—C22	1.377 (3)
C6—C7	1.383 (3)	C21—H21	0.9300
C6—H6	0.9300	C22—C23	1.372 (3)
C7—C8	1.375 (3)	C22—H22	0.9300
C8—C9	1.380 (3)	C23—N14	1.317 (3)
C8—H8	0.9300	C23—Cl2	1.750 (2)
C9—C10	1.368 (3)	N3—H3A	0.8600
C9—H9	0.9300	N4—N5	1.362 (2)
C10—N7	1.310 (3)	N5—N6	1.315 (2)
C10—Cl1	1.753 (2)	N10—H10A	0.8600
C11—N2	1.477 (2)	N11—N12	1.364 (2)
C11—C12	1.478 (3)	N12—N13	1.318 (2)
C11—H11A	0.9700		
			
C25—C24—N9	114.8 (2)	H13A—C13—H13B	120.0
C25—C24—H24A	108.6	N13—C14—C17	109.22 (16)
N9—C24—H24A	108.6	N13—C14—C15	129.91 (16)
C25—C24—H24B	108.6	C17—C14—C15	120.84 (16)
N9—C24—H24B	108.6	N10—C15—N9	119.50 (18)
H24A—C24—H24B	107.6	N10—C15—C14	130.94 (18)
C26—C25—C24	129.2 (4)	N9—C15—C14	109.56 (15)
C26—C25—H25	115.4	N8—C16—N9	127.79 (18)
C24—C25—H25	115.4	N8—C16—H16	116.1
C25—C26—H26A	120.0	N9—C16—H16	116.1
C25—C26—H26B	120.0	N11—C17—N8	127.49 (16)
H26A—C26—H26B	120.0	N11—C17—C14	104.94 (15)
N6—C1—C4	109.19 (15)	N8—C17—C14	127.56 (17)
N6—C1—C2	129.88 (15)	N11—C18—C20	113.36 (16)
C4—C1—C2	120.92 (15)	N11—C18—H18A	108.9
N3—C2—N2	119.22 (16)	C20—C18—H18A	108.9
N3—C2—C1	131.28 (16)	N11—C18—H18B	108.9
N2—C2—C1	109.50 (14)	C20—C18—H18B	108.9
N1—C3—N2	127.88 (17)	H18A—C18—H18B	107.7
N1—C3—H3	116.1	N14—C19—C20	124.30 (18)
N2—C3—H3	116.1	N14—C19—H19	117.8
N4—C4—N1	127.68 (16)	C20—C19—H19	117.8
N4—C4—C1	104.78 (15)	C19—C20—C21	116.96 (19)
N1—C4—C1	127.53 (17)	C19—C20—C18	122.96 (17)
N4—C5—C7	110.31 (14)	C21—C20—C18	120.06 (18)
N4—C5—H5A	109.6	C22—C21—C20	119.9 (2)
C7—C5—H5A	109.6	C22—C21—H21	120.0
N4—C5—H5B	109.6	C20—C21—H21	120.0
C7—C5—H5B	109.6	C23—C22—C21	117.6 (2)
H5A—C5—H5B	108.1	C23—C22—H22	121.2
N7—C6—C7	124.30 (18)	C21—C22—H22	121.2
N7—C6—H6	117.8	N14—C23—C22	125.2 (2)
C7—C6—H6	117.8	N14—C23—Cl2	115.96 (17)
C8—C7—C6	117.12 (18)	C22—C23—Cl2	118.84 (17)
C8—C7—C5	122.66 (18)	C3—N1—C4	110.65 (15)
C6—C7—C5	120.18 (17)	C3—N2—C2	123.51 (15)
C7—C8—C9	119.8 (2)	C3—N2—C11	118.39 (15)
C7—C8—H8	120.1	C2—N2—C11	118.09 (15)
C9—C8—H8	120.1	C2—N3—H3A	119.3
C10—C9—C8	117.16 (19)	C4—N4—N5	110.08 (14)
C10—C9—H9	121.4	C4—N4—C5	129.06 (15)
C8—C9—H9	121.4	N5—N4—C5	120.63 (15)
N7—C10—C9	125.59 (19)	N6—N5—N4	107.99 (14)
N7—C10—Cl1	115.58 (16)	N5—N6—C1	107.95 (14)
C9—C10—Cl1	118.82 (16)	C10—N7—C6	115.93 (17)
N2—C11—C12	113.26 (16)	C16—N8—C17	110.70 (16)
N2—C11—H11A	108.9	C16—N9—C15	123.52 (17)
C12—C11—H11A	108.9	C16—N9—C24	120.41 (17)
N2—C11—H11B	108.9	C15—N9—C24	116.08 (16)
C12—C11—H11B	108.9	C15—N10—H10A	119.3
H11A—C11—H11B	107.7	C17—N11—N12	109.97 (14)
C13—C12—C11	124.9 (3)	C17—N11—C18	129.37 (16)
C13—C12—H12	117.5	N12—N11—C18	120.66 (16)
C11—C12—H12	117.5	N13—N12—N11	107.63 (15)
C12—C13—H13A	120.0	N12—N13—C14	108.23 (15)
C12—C13—H13B	120.0	C23—N14—C19	116.03 (18)
			
N9—C24—C25—C26	−120.5 (5)	N3—C2—N2—C11	0.2 (2)
N6—C1—C2—N3	0.1 (3)	C1—C2—N2—C11	179.95 (14)
C4—C1—C2—N3	−179.05 (17)	C12—C11—N2—C3	−89.6 (2)
N6—C1—C2—N2	−179.57 (15)	C12—C11—N2—C2	89.2 (2)
C4—C1—C2—N2	1.3 (2)	N1—C4—N4—N5	−179.62 (15)
N6—C1—C4—N4	−0.42 (17)	C1—C4—N4—N5	0.66 (17)
C2—C1—C4—N4	178.88 (14)	N1—C4—N4—C5	−5.2 (3)
N6—C1—C4—N1	179.86 (15)	C1—C4—N4—C5	175.05 (15)
C2—C1—C4—N1	−0.8 (3)	C7—C5—N4—C4	−101.8 (2)
N7—C6—C7—C8	2.9 (3)	C7—C5—N4—N5	72.1 (2)
N7—C6—C7—C5	−175.14 (18)	C4—N4—N5—N6	−0.68 (18)
N4—C5—C7—C8	−105.4 (2)	C5—N4—N5—N6	−175.62 (14)
N4—C5—C7—C6	72.5 (2)	N4—N5—N6—C1	0.39 (17)
C6—C7—C8—C9	−1.9 (3)	C4—C1—N6—N5	0.02 (18)
C5—C7—C8—C9	176.1 (2)	C2—C1—N6—N5	−179.19 (15)
C7—C8—C9—C10	−0.5 (3)	C9—C10—N7—C6	−1.6 (3)
C8—C9—C10—N7	2.4 (4)	Cl1—C10—N7—C6	177.57 (15)
C8—C9—C10—Cl1	−176.73 (18)	C7—C6—N7—C10	−1.2 (3)
N2—C11—C12—C13	123.7 (3)	N9—C16—N8—C17	−0.1 (3)
N13—C14—C15—N10	−0.3 (4)	N11—C17—N8—C16	179.08 (19)
C17—C14—C15—N10	177.7 (2)	C14—C17—N8—C16	−0.8 (3)
N13—C14—C15—N9	−179.85 (18)	N8—C16—N9—C15	−0.3 (3)
C17—C14—C15—N9	−1.9 (2)	N8—C16—N9—C24	179.6 (2)
N13—C14—C17—N11	0.4 (2)	N10—C15—N9—C16	−178.4 (2)
C15—C14—C17—N11	−178.00 (16)	C14—C15—N9—C16	1.2 (3)
N13—C14—C17—N8	−179.76 (18)	N10—C15—N9—C24	1.6 (3)
C15—C14—C17—N8	1.9 (3)	C14—C15—N9—C24	−178.73 (18)
N14—C19—C20—C21	0.7 (3)	C25—C24—N9—C16	5.5 (4)
N14—C19—C20—C18	179.01 (18)	C25—C24—N9—C15	−174.5 (3)
N11—C18—C20—C19	27.4 (3)	N8—C17—N11—N12	179.79 (18)
N11—C18—C20—C21	−154.32 (18)	C14—C17—N11—N12	−0.3 (2)
C19—C20—C21—C22	0.1 (3)	N8—C17—N11—C18	−0.3 (3)
C18—C20—C21—C22	−178.26 (19)	C14—C17—N11—C18	179.60 (18)
C20—C21—C22—C23	−0.6 (3)	C20—C18—N11—C17	−110.6 (2)
C21—C22—C23—N14	0.4 (3)	C20—C18—N11—N12	69.3 (2)
C21—C22—C23—Cl2	−179.65 (16)	C17—N11—N12—N13	0.2 (2)
N2—C3—N1—C4	0.0 (3)	C18—N11—N12—N13	−179.76 (17)
N4—C4—N1—C3	−179.58 (17)	N11—N12—N13—C14	0.1 (2)
C1—C4—N1—C3	0.1 (2)	C17—C14—N13—N12	−0.3 (2)
N1—C3—N2—C2	0.6 (3)	C15—C14—N13—N12	177.90 (19)
N1—C3—N2—C11	179.45 (18)	C22—C23—N14—C19	0.3 (3)
N3—C2—N2—C3	179.06 (16)	Cl2—C23—N14—C19	−179.61 (14)
C1—C2—N2—C3	−1.2 (2)	C20—C19—N14—C23	−0.9 (3)

### Hydrogen-bond geometry (Å, °)

**Table:**

*D*—H···*A*	*D*—H	H···*A*	*D*···*A*	*D*—H···*A*
N10—H10A···N1	0.86	2.44	3.292 (3)	173
N3—H3A···N8^i^	0.86	2.45	3.299 (2)	169
C11—H11A···N3	0.97	2.41	2.749 (2)	100
C19—H19···N11	0.93	2.59	2.909 (3)	101
C3—H3···N13	0.93	2.46	3.309 (2)	151
C11—H11A···Cg4^ii^	0.97	2.87	3.446 (2)	119
C24—H24A···Cg1^iii^	0.97	2.99	3.851 (3)	149

Symmetry codes: (i) *x*, *y*+1, *z*; (ii) −*x*+1, −*y*+2, −*z*+1; (iii) −*x*+2, −*y*+1, −*z*+1.
